# Non-inactivating voltage-activated K^+^ conductances can increase photoreceptor signaling bandwidth beyond the bandwidth set by phototransduction

**DOI:** 10.1371/journal.pone.0289466

**Published:** 2023-08-01

**Authors:** Roman V. Frolov

**Affiliations:** Laboratory of Comparative Sensory Physiology, Sechenov Institute of Evolutionary Physiology and Biochemistry, Russian Academy of Sciences, Saint Petersburg, Russia; University of Leipzig Faculty of Life Sciences: Universitat Leipzig Fakultat fur Lebenswissenschaften, GERMANY

## Abstract

Evolution produced a large variety of rhabdomeric photoreceptors in the compound eyes of insects. To study effects of morphological and electrophysiological differences on signal generation and modulation, we developed models of the cockroach and blow fly photoreceptors. The cockroach model included wide microvilli, large membrane capacitance and two voltage-activated K^+^ conductances. The blow fly model included narrow microvilli, small capacitance and two sustained voltage-activated K^+^ conductances. Our analysis indicated that membrane of even the narrowest microvilli of up to 3 μm long can be measured fully from the soma. Attenuation of microvillar quantum bump (QB)-like signals at the recording site in the soma increased with the signal amplitude in the microvillus, due to the decreasing driving force. However, conductance of the normal-sized QBs can be detected in the soma with minimal attenuation. Next, we investigated how interactions between the sustained voltage-activated K^+^ and light-induced conductances can shape the frequency response. The models were depolarized by either a current injection or light-induced current (LIC) and probed with inward currents kinetically approximating dark- or light-adapted QBs. By analyzing the resulting voltage impulse responses (IR), we found that: (1) sustained K^+^ conductance can shorten IRs, expanding the signaling bandwidth beyond that set by phototransduction; (2) voltage-dependencies of changes in IR durations have minima within the physiological voltage response range, depending on the activation kinetics of K^+^ conductance, the presence or absence of sustained LIC, and the kinetics of the probing current stimulus; and (3) sustained LIC lowers gain of IRs and can exert dissimilar effects on their durations. The first two findings were supported by experiments. It is argued that improvement of membrane response bandwidth by parametric interactions between passive, ligand-gated and voltage-dependent components of the membrane circuit can be a general feature of excitable cells that respond with graded voltage signals.

## Introduction

The most salient feature of rhabdomeric photoreceptors from compound eyes of insects is their microvilli arranged in compact three-dimensional arrays. Rhabdoms vary greatly between terrestrial insect species in the numbers and dimensions of microvilli [1, 2]. The number of microvilli can reach hundreds of thousands in photoreceptors of large diurnal flies or nocturnal cockroaches. The length of the microvillus depends, among other things, on the visual ecology of the species, the spectral class and location of the photoreceptor in the eye [2]. For example, in the frontal-dorsal part of the compound eye of the hover fly *Volucella pellucens*, the microvillus diameter is ~40 nm and length ~1000 nm, with only about ten microvilli of similar length per cross-section of the rhabdomere [3]. In contrast, in the eye of the crepuscular American cockroach *Periplaneta americana*, the microvillus diameter is ~100 nm, its length varies from hundreds of nanometers to several micrometers, and the number per cross-section can exceed one hundred [2, 4]. Microvilli of big diurnal flies appear to be irreducibly small for a standalone cellular compartment because the diameter of <40 nm accommodates ~7 nm of plasma membrane and a similar measure of the central filament. Currently, there is no evolutionary rationale for the narrowing of the microvillus in the big flies, and for the variability of the microvillus diameter between species.

Depending on the number and dimensions of the microvilli, the area of the rhabdomeric membrane can be comparable or even exceed the area of the light-insensitive membrane. Containing no voltage-activated conductances, rhabdomeric membrane can heavily contribute to the low-pass filtering of transient quantum bumps (QBs) generated by the phototransduction cascade. However, microvilli are cable structures that can have aspect ratios of up to 50:1 (see images in [5, 6]), with a short neck about two times narrower than the body. The membrane resistance in the microvillus decreases when it is activated by light and transduction channels open shortening the length constant. This raises several questions motivating this study:

1. How can the microvillus topology and its variability between species affect the visibility of the microvillus membrane in the soma, i.e. to what extent does the microvillus membrane contribute to *C*_m_, when *C*_m_ is measured in the soma? By “visibility” of a parameter we mean a fraction of the true measure of a peripheral quantity, such as the membrane area of microvillus or QB conductance evoked therein, that can be measured using recordings in the soma [7]. The mismatch between the measured and true values is due to non-isopotentiality of extended cable-like cellular structures. In the previous studies, we discovered significant mechanistic correlations between photoreceptor membrane areas and several functional properties [8–10] (see Discussion). Membrane areas can be estimated using different methodologies, in intracellular recordings with a sharp microelectrode from intact animals, or in whole-cell patch-clamp recordings from photoreceptors in dissociated ommatidia. All such recordings are performed from the soma, and in the absence of information about the cable properties of the rhabdomere the functional significance of the correlations remains uncertain.
2. What effect does the microvillus geometry have on light-induced signals evoked therein? Like the size of the microvillus, the QB size as recorded in the soma varies both within and between insect species [2, 11]. Since recording of QBs directly from the microvillus seem to be unfeasible, the only approach to study QBs at their source is experimentation *in silico*.
3. What is the contribution of microvillar conductance to that of the soma? Two major models of fly photoreceptors that can generate voltage responses to light stimulation have been published previously [12–15]. The main difference between their equivalent electrical circuits is the lack of light-induced conductance in the model by Song and Juusola [14]. It is essential to determine whether or not such omission can affect the simulated light responses.
4. How do voltage-activated K^+^ conductances modulate voltage responses to light?
5. How does sustained light-induced conductance modulate the voltage responses?

Two last questions touch a complex problem of interactions between the voltage-driving light-induced conductance, passive electrical parameters of the membrane (*C*_m_ and input resistance), and the voltage-activated K^+^ conductances. It is well-known that in insect photoreceptors K^+^ conductances can profoundly modify the primary receptor signal generated by the phototransduction cascade when it is transformed into a voltage response, with the outcome depending on the kinetic properties of K^+^ conductances [12, 16–19]. Arguably, this problem is not limited to the rhabdomeric photoreceptors but universal for the excitable cells. In the receptor cells of sensory systems, the external input signal is usually transduced into a depolarizing voltage transient by ion channels gated by ligands or directly by input’s energy [20]. Further transmission of such graded voltage signals, often involving their transformation into action potentials, can be influenced by voltage-activated K^+^ conductances. Likewise, in neurons, transient excitatory potentials generated by depolarizing postsynaptic currents via ligand-gated receptor channels can be significantly modulated by voltage-activated K^+^ conductances [21, 22].

Here, we developed a topological model of the rhabdomeric photoreceptor in NEURON. Then, by modifying dimensions of the microvilli, passive electrical properties of the membrane, kinetics and densities of voltage-activated K^+^ conductances, we simulated photoreceptors of the cockroach *P*. *americana* and the blow fly *Calliphora vicina*. Investigation of the questions listed above revealed that: (1) the rhabdomeric membrane is fully visible from the soma; (2) the normal-sized QB-like signals from the microvillus are almost not attenuated; (3) the conductance underlying the QB-like signals in the microvillus adds almost completely to the somatic conductance; and (4) the sustained voltage-activated K^+^ conductance can exert strong non-linear effects on voltage responses increasing the signaling bandwidth, whereas effects of the background light-induced conductance are complex and parametric.

## Materials and methods

### Electrophysiology and data analysis

We used electrophysiological data from two previously published datasets [3, 23].

The signal gain function *T*_xy_(*f*) was calculated by dividing the cross-spectrum of the photoreceptor input (a naturalistic or white-noise modulated light stimulus) and output (photoreceptor voltage or current response) *P*_xy_(*f*), by the auto-spectrum of the input *P*_xx_(*f*) and taking the absolute value of the resulting frequency response function:

$$T_{xy}(f)=\frac{P_{xy}(f)}{P_{xx}(f)}$$

The corner frequency was defined as the frequency at which signal gain decreased by 50%. It was determined by fitting the gain function in the 1–200 Hz range with a Hill equation.

### Modeling in NEURON

Neuron 7.8 was used for modeling. Tables 1 and 2 list geometric and electrophysiological parameters of the models.

**Table 1:** Geometry and conductances of the cockroach model.

Compartment	length, μm	diameter, μm	*g*_pas_, mS cm^-2^	*I*_A_, mS cm^-2^	*I*_DR_, mS cm^-2^
soma	300	30	0.05	0.25	0.5
microvillus	1	0.1	0.05	0	0
microvillus neck	0.05	0.05	0.01	0	0
axon	120	1	0.05	0	0

**Table 2:** Geometry and conductances of the blow fly model.

Compartment	length, μm	diameter, μm	*g*_pas_, mS cm^-2^	*I*_KF_, mS cm^-2^	*I*_KS_, mS cm^-2^
soma	300	12	0.15	0.4	0.1
microvillus	1	0.04	0.05	0	0
microvillus neck	0.05	0.02	0.01	0	0
axon	120	1	0.15	0.4	0

In both models, the number of microvilli was 31. The number of segments was 10 for the microvillus, soma and axon, allowing to place 10 point sources in each compartment. Reversal potential *E*_pas_ was -60 mV, and *E*_K_ -77 mV. Specific resistance of cytoplasm was 35.4 Ω∙cm and specific membrane capacitance 1 μF cm^-2^.

Voltage-activated K^+^ conductances were modeled using the Hodgkin-Huxley formalism. Non-inactivating K^+^ channel subunit opening probability was

$$\frac{dn}{dt}=a_{n}∙(1-n)-b_{n}∙n$$

The activation rate *a*_n_ was

$$a_{n}=\frac{A∙k_{a}∙(V_{m}-V_{a})}{1-exp(-k_{a}∙(V_{m}-V_{a}))}$$

where *A* describes the rate of subunit opening, *k*_a_ the voltage dependence, and *V*_a_ the half-activation potential. The deactivation rate *b*_n_ was

$$b_{n}=B∙{exp(k}_{b}∙(V_{m}-V_{b}))$$

where *B* describes the rate of subunit closing, *k*_b_ the voltage dependence, and *V*_b_ the half-deactivation potential. The resulting sustained current was

$$I_{K}(t)=g_{K}∙n^{4}∙(V_{m}-E_{K}))$$

To model the transient voltage-activated K^+^ conductance *I*_A_, in addition to the activation term *n* described above, inactivation *h* was introduced so that

$$\frac{dh}{dt}=a_{h}∙(1-h)-b_{h}∙h$$

where the recovery from inactivation rate *a*_h_ was

$$a_{h}=A∙exp(k_{a}∙(V_{m}-V_{a}))$$

and inactivation rate *b*_h_

$$b_{h}=\frac{B}{1+exp(-k_{b}∙(V_{m}-V_{b}))}$$

The overall current was

$$I_{A}(t)=g_{K}∙n^{3}∙h∙(V_{m}-E_{K}))$$

All parameters used to simulate K^+^ currents are listed in Table 3.

**Table 3:** Parameters of K^+^ conductances used in the models.

K^+^ current	Activation rate *a*_n_			Deactivation rate *b*_n_		
	*A*, ms^-1^	*k*_a_, mV^-1^	*V*_a_, mV	*B*, ms^-1^	*k*_b_, mV^-1^	*V*_b_, mV
blow fly *I*_KF_	0.2	0.08	-55	0.2	-0.03	-100
blow fly *I*_KS_	0.045	0.15	-42	0.04	-0.022	-140
cockroach *I*_DR_	0.06	0.06	-35	0.1	-0.032	-90
cockroach *I*_A_	0.4	0.08	-44	1	-0.056	-80
	Recovery from inactivation rate *a*_h_			Inactivation rate *b*_h_		
	0.07	-0.05	-85	0.2	-0.1	-30

## Results

### Visibility of the rhabdomeric membrane from the soma

Because the area of the light-sensitive rhabdomeric membrane usually comprises a large fraction of the total photoreceptor membrane area, the extent of low-pass filtering of light responses in the soma depends on the visibility of the microvillar membrane.

We investigated visibility of membrane encompassing microvilli of two diameters and different lengths. The diameters, 40 and 100 nm, delimit the empirically determined range of microvillar diameter variability in species with rhabdomeric photoreceptors. For example, the rhabdom of the crepuscular cockroach *P*. *americana* contains a large number of thick (~100 nm in diameter) microvilli [4]. In contrast, the rhabdom cross-section in the highly aerobatic diurnal muscoid fly *Lucilia cuprina* contains ~25 short and narrow (~40 nm in diameter) microvilli [24]. Although the narrowest microvilli, with diameters of ≤40 nm, are usually found in fast diurnal flies, no evolutionary pattern linking microvillar dimensions to the properties of QBs they generate can be discerned. For example, microvilli in the photoreceptors of agile diurnal butterflies have about the same diameters as in the cockroach (~90 nm) [25].

**Fig 1:**
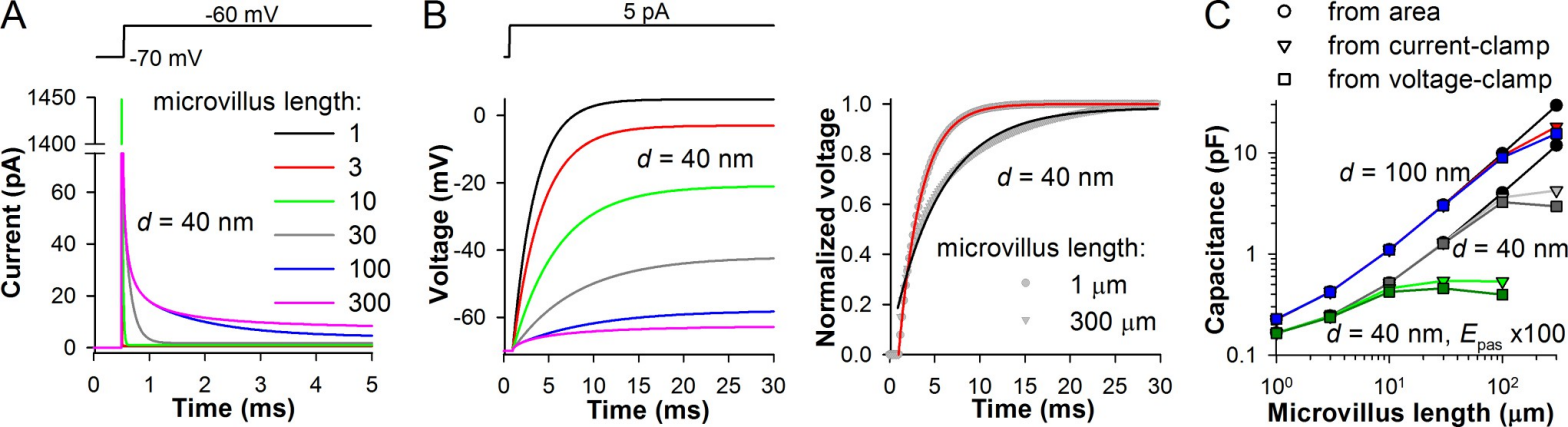
Visibility of the rhabdomeric membrane from the soma and membrane capacitance. Models with a small passive soma and 31 microvilli of different lengths and two diameters (40 and 100 nm) were used to evaluate the effects of microvillus geometry on the visibility of the rhabdom from the soma. Microvillus neck diameter was 50% of the microvillus diameter, with the length fixed at 50 nm. Cm values were obtained in voltage- (A) and current-clamp (B) simulations and compared to those calculated from the total membrane area AM (AM multiplied by specific capacitance of 1 μF/cm2). (A) In the voltage-clamp simulations, the model cell was perturbed by a 50-ms 10-mV pulse from a holding potential of -70 mV; Cm was measured by dividing the area under the capacitive transient with the voltage step amplitude; in the case of 100 and 300 μm-long microvilli, the area under the slow component of the capacitive transient was also integrated. (B) In the current-clamp simulations, Cm was measured from the membrane time constant; τm was determined by fitting the voltage response onset with a single exponential equation. Left: voltage responses of different models to a 5 pA current pulse. Right: comparison of voltage responses of an isopotential (1 μm microvillus) and non-isopotential (300 μm microvillus) models. (C) Comparison of Cm values obtained by three methods for the models with different microvillus diameters (100 and 40 nm), and a 40-nm model with a 100-fold higher passive conductance of the microvillus membrane.

To determine how microvillar dimensions influence the visibility of the rhabdomeric membrane, we compared the membrane area of the model (*A*_M_) with that derived from capacitance (*C*_m_) estimates obtained in current- and voltage-clamp simulations (Fig 1). In the voltage-clamp mode, *C*_m_ can be found by dividing the area under the capacitive transient elicited by a step-like change in membrane potential with the voltage step size (Fig 1A). In the current-clamp mode, *C*_m_ can be obtained from the membrane time constant (τ_m_) of the response onset, by dividing τ_m_ with the associated membrane resistance (*R*_m_) value (Fig 1B).

However, membrane area of a single microvillus is usually very small compared to the area of the soma. If cable properties of the microvillus lower its membrane visibility only slightly, then in order to detect a small difference between *A*_M_ and membrane area estimates from *C*_m_, the total membrane area of the rhabdom must be sufficiently large. However, since we were able to develop a stable model with 31 microvilli only, to increase the relative size of the rhabdomeric membrane, the size of the soma had to be reduced.

Therefore, all 31 microvilli were attached via their necks to a small cylindrical soma (diameter 2 μm, length 2 μm) at point 0. In the model with microvilli that were 1 μm long and 100 nm in diameter, *A*_M_ of the soma was 12.56 μm^2^ and *A*_M_ of the rhabdom 9.98 μm^2^, totaling 22.53 μm^2^, which corresponds to *C*_m_ of 0.225 pF. The model was completely passive, with the evenly distributed passive membrane conductance (*g*_pas_) of 0.1 mS cm^-2^. This conductance density value lies in-between the empirically determined passive conductances of the blow fly and cockroach photoreceptors (see below). When this model was perturbed in the middle of the soma with a 10 mV step in the single-electrode voltage-clamp (SEVC) mode, *C*_m_ measured from the capacitive transient equaled 0.228 pF. Similarly, *C*_m_ measured in the current-clamp mode using a current pulse was 0.227 pF.

In Fig 1A and 1B, a model with the narrow (40 nm) microvilli, the lengths of which increased from 1 to 300 μm, was tested in SEVC and current-clamp simulations. In the models with microvilli of up to 30 μm long, the capacitive transients in the SEVC mode settled within a fraction of millisecond (Fig 1A). Likewise, in the current-clamp mode, the onsets of the corresponding curves could be well fitted with a single-exponential equation. These results indicate that the system’s response to the signal from the soma was not distorted significantly by the length constant of the microvilli. However, when the microvillus was elongated first to 100 and then to 300 μm, the capacitive transients acquired a slow component (Fig 1A), and the onsets of voltage responses could no longer be fitted with a single-exponential equation (Fig 1B, right), indicating that the models were not isopotential.

The summary of experiments in the small-soma model is presented in Fig 1C. The two microvillus diameters we used, 40 and 100 nm, simulate microvilli of the blow fly and cockroach, respectively. In the cockroach model, three *C*_m_ values (from *A*_M_, SEVC and current-clamp) matched up to the microvillus length of 100 μm, above which the experimentally determined *C*_m_ values started to become smaller than the *A*_M_-derived *C*_m_. In the blow fly model, deviation from the isopotentiality was observed above the length of 30 μm, due to comparatively narrow microvilli.

Because the length constant depends on membrane resistance, it was also necessary to test how an increase in membrane conductance can affect the visibility of the rhabdomeric membrane. Such increase simulates opening of transduction channels in the microvilli and lowers the length constant. This was tested in the blow fly model. Fig 1C shows that upon a 100-fold decrease in passive membrane resistance in the microvilli the model remained isopotential up to the microvillus length of 3 μm. Because microvilli in photoreceptors of insects rarely exceed 3 μm, our modeling results suggest that rhabdomeric membrane fully contributes to *C*_m_ that shapes voltage responses in the soma, even when the microvillus is activated and generates a QB.

Another factor that can affect the visibility of the rhabdomeric membrane is the neck of the microvillus. All microvilli are connected to the soma via narrow short necks, which are about two times narrower than the microvillus itself and have a length of ~50 nm. We varied the neck diameter systematically but effects on *C*_m_ were observed only when it was lowered to single nanometers.

### Modeling the light-insensitive membrane of *P*. *americana* photoreceptor

**Fig 2:**
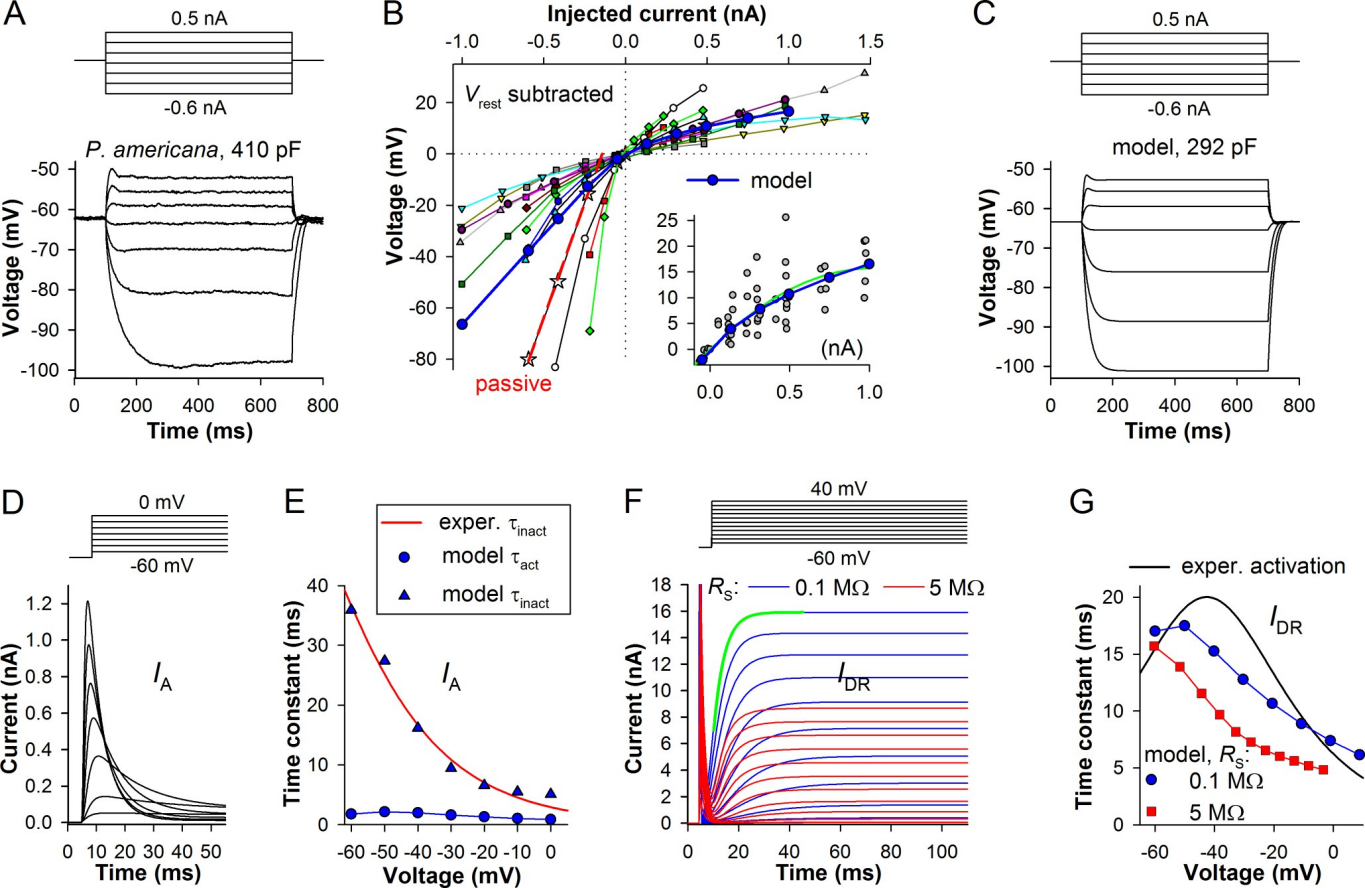
Modeling electrical properties of the light-insensitive membrane of *P*. *americana*. (A) A typical in-vivo current-clamp recording from a cockroach photoreceptor. (B) Changes in sustained (e.g. between 400 and 600 ms as in A) membrane potential caused by depolarizing or hyperpolarizing current pulses in 15 photoreceptors in vivo (small symbols connected by thin lines) and in the modeled photoreceptor; resting potentials were subtracted. Red dashed line: an example of determining passive membrane resistance (see Results). Inset: all experimental values elicited by depolarizing pulses up to 1 nA (gray circles) were pooled together and fitted with a polynomial equation (green trace); the voltage-current relationship from the model (blue trace) closely follows the fitting trace. (C) A current-clamp recording in the model using the same stimulation protocol as in A. (D, E) Model of the transient voltage-activated K^+^ current (*I*_A_, D) was obtained by adjusting model parameters so that the resulting time constants of activation and inactivation (E) in the voltage range from -60 to 0 mV were similar to the previously published values [26]; the SEVC recording was simulated using series resistance (*R*_s_) of 0.1 MΩ. (F) Sustained voltage-activated K^+^ current (*I*_DR_) was simulated using two series resistance values as indicated. (G) Dependencies of *I*_DR_ activation time constants on the experimental conditions; values from our model are compared to those from the previous study [26]; to obtain *I*_DR_ activation time constants in the model, the second half of *I*_DR_ onset was fitted with a single exponential equation (e.g. green trace in F); voltage errors were corrected.

An electrical model of the light-insensitive (somatic) membrane must reproduce the experimentally derived voltage responses to current injections. First, we developed a cockroach photoreceptor model. A typical current-clamp recording is shown in Fig 2A. The voltage responses have three features. When the cell is driven into a hyperpolarized state by a current injection (*I*_inj_), its voltage response becomes passive. By fitting the response onset slope in the passive range with a single-exponential equation and dividing the resulting τ_m_ with the associated *R*_m_ value (where *R*_m_ = Δ*V*_m_/*I*_inj_, and Δ*V*_m_ is the settled voltage difference caused by the current pulse), *C*_m_ can be obtained (*C*_m_ = τ_m_/*R*_m_). Membrane resistance in the passive range can be used to obtain *g*_pas_. When membrane is depolarized above the resting potential (*V*_rest_), sustained (non-inactivating) delayed rectifier K^+^ conductance (*I*_DR_) provides negative feedback, curtailing depolarization. Depending on the activation rate of *I*_DR_ channels and/or presence of a fast-activating, usually transient K^+^ conductance (*I*_A_), the size of the initial voltage transient can vary. Therefore, to model photoreceptor’s response in the dark, one needs the average *C*_m_ and *g*_pas_ values, and also the densities and kinetic parameters of voltage-activated conductances.

Cockroach photoreceptors vary strongly in their electrophysiological properties [11]. The cells are large compared to photoreceptors of other insects [2], with a median *C*_m_ of 363 pF for broadband photoreceptors [4]. However, the peak of *C*_m_ distribution is at 300 pF, and so the membrane area of the cockroach photoreceptor model was made to have a similar size.

Importantly, because we could not reproduce the rhabdomere consisting of tens of thousands microvilli, nearly all membrane had to be allocated to the soma. Therefore, dimensions of the soma were made larger than in vivo. The length of the cockroach ommatidium is normally about 250–350 μm [27]. At the cross-sections, photoreceptor somas have diameters in the range of 5–10 μm [27]. The somas appear to be arranged in two tiers [28]. Thus instead of a cell of ~150 μm in length and ~7 μm in diameter, the soma in the model was a 300-μm long cylinder with a diameter of 30 μm (Table 1).

The variability in voltage-current relationships reflecting that of the voltage-dependence of *R*_m_ in the dark is illustrated in Fig 2B using data from 15 photoreceptors. For presentation purposes, *V*_rest_ values were subtracted. These data were used to obtain *g*_pas_ and *I*_DR_ conductance values for the model. To estimate *g*_pas_, the settled *V*_m_ values of two or three most hyperpolarized responses were fitted with a linear equation (Fig 2B, e.g. red dashed line). Median *g*_pas_ was 64(37:178) MΩ. In the model, we distributed *g*_pas_ evenly over the surface of the soma, microvilli and axon, so that for a model cell with *C*_m_ of 292 pF the *g*_pas_ density was 5∙10^−5^ S cm^-2^ (Table 1). Fig 2C shows responses of the model to the same current stimulation protocol as in Fig 2A. Inset in Fig 2B demonstrates that sustained depolarized voltage responses of the model coincide with the experimental trend line.

To model the typical cockroach photoreceptor responses to current pulses, we introduced two K^+^ conductances, *I*_A_ and *I*_DR_ (Fig 2D–2G). The simulated *I*_A_ is shown in Fig 2D. It was reconstructed using kinetic and conductance data from the previous study [26]. *I*_A_ was implemented using the Hodgkin-Huxley formalism by adjusting parameters of activation, deactivation, inactivation and recovery from inactivation (Table 3). Fig 2E shows voltage-dependencies of two parameters that were most important for our model, the time constants of activation (τ_act_) and inactivation (τ_inact_), which can be obtained by fitting the current responses with an exponential product equation

$$I(t)=A\left(1-e^{-\frac{t}{\tau_{act}}}\right)\left(e^{-\frac{t}{\tau_{inact}}}\right)+C.$$

In the study by Salmela et al. *I*_A_ activation time constants could not be measured due to superposition of the capacitive transient and the activating *I*_DR_. They were modeled to be voltage-independent at the level of 1 ms [26]. However, inactivation time constants were comparatively large, and we matched them well in the model (Fig 2E). Amplitude-wise, *I*_A_ in the patch-clamp experiments was quite small compared to *I*_DR_, and totally undetectable in a large fraction of cells. In the cockroach model, *I*_A_ *g*_max_ was set twice as small as *g*_max_ for *I*_DR_ (Table 1).

In the cockroach, *I*_DR_ is mainly mediated by EAG channels, with a possible contribution of KCNQ [19]. *I*_DR_ varies strongly between the cells, both in activation kinetics and amplitudes [26]. In the model, we used the *I*_DR_ shown in Fig 2F. Two sets of traces illustrate the effect of series resistance (*R*_S_) on *I*_DR_ in a SEVC experiment. All information about cockroach *I*_DR_ was acquired in vitro, using patch-clamp recordings from photoreceptors in dissociated ommatidia. In such experiments, pipette resistance is at least 3 MΩ and the whole-cell *R*_S_ as a rule exceeds 10 MΩ before the compensation. In practice, at best, *R*_S_ could not be compensated to <5 MΩ. It can be seen that the residual *R*_S_ strongly alters the current responses. Firstly, capacitive transient is widened because capacitance is charged relatively slowly via *R*_S_. Secondly, voltage error progressively lowers sustained current amplitudes. Thirdly, the apparent τ_act_ diminishes because voltage error increases dynamically during the onset of the current, accelerating it.

As in the previous study [26], we determined τ_act_ values by fitting the second halves of *I*_DR_ onsets with a single-exponential equation (Fig 2F, green trace). Fig 2G compares τ_act_ values from Fig 2F with experimental data (black trace). Because experimental data contain a series resistance error, patch-clamp results need to be compared with τ_act_ values obtained in SEVC simulation with *R*_S_ of 5 MΩ. The modeled *I*_DR_ is characterized by a faster activation kinetics than the experimental *I*_DR_. This deviation from the experimental results was dictated by a necessity to reproduce the initial depolarizing transient characterizing the in-vivo current-clamp response (Fig 2A). If *I*_DR_ activation kinetics of the patch-clamp data was used, the transient would become tens millivolts high and tens millisecond wide, even if a much larger *I*_A_ was introduced. We have previously demonstrated that recordings from dissociated ommatidia are characterized by comparatively slow and wide responses to light [29]. It is possible that dissociation procedure or in-vitro recording conditions not only deteriorate the phototransduction cascade but also slow the gating of EAG channels, which are regulated via several molecular mechanisms [19].

### Modeling the light-insensitive membrane of *C*. *vicina* photoreceptor

**Fig 3:**
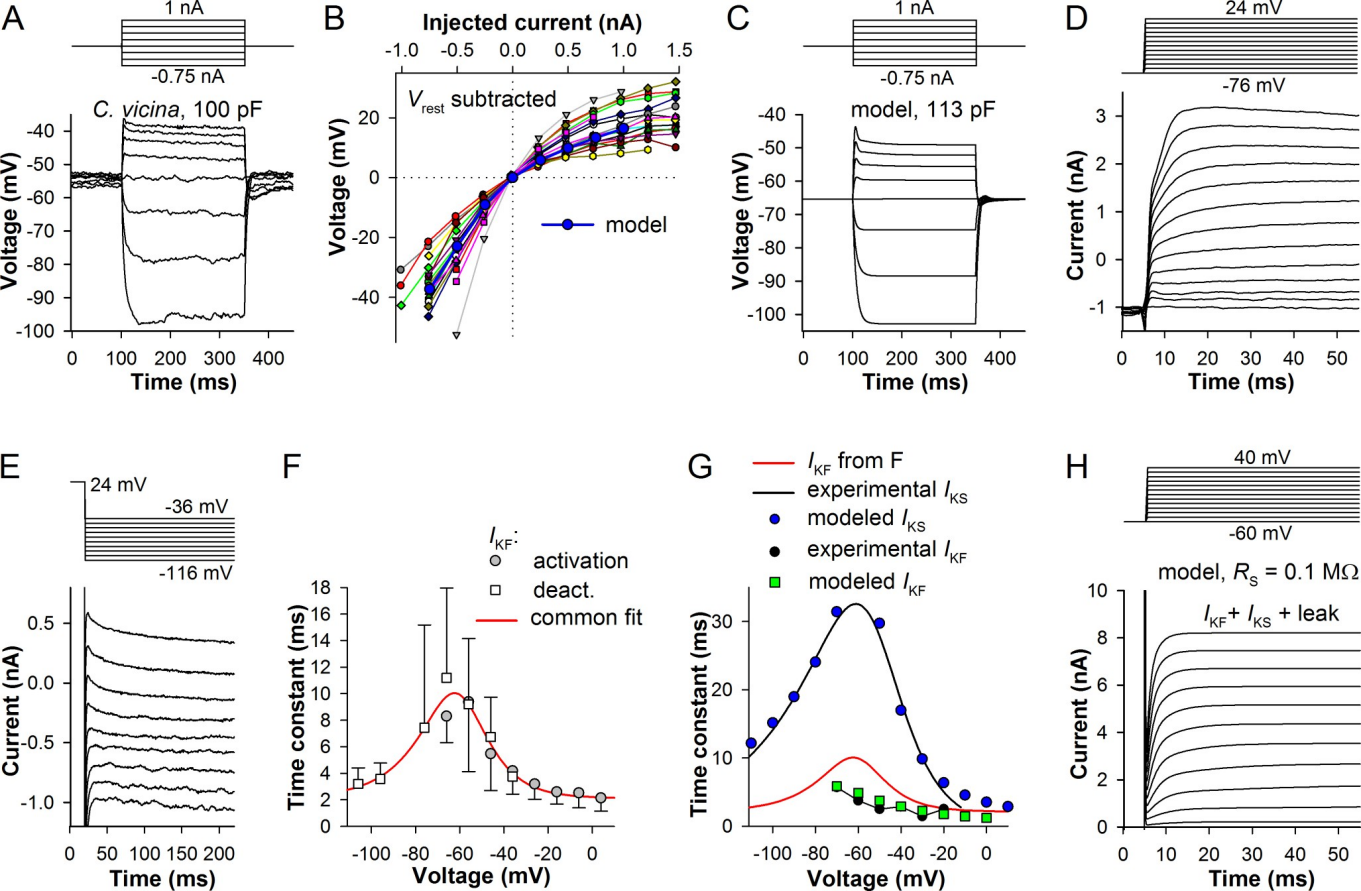
Modeling electrical properties of the light-insensitive membrane of *C*. *vicina*. (A) A typical in-vivo current-clamp recording from a blowfly photoreceptor. (B) Changes in sustained (e.g. between 250 and 350 ms as in A) membrane potential caused by depolarizing or hyperpolarizing current pulses in 20 photoreceptors (small symbols connected by thin lines); resting potentials were subtracted; thick blue trace is the voltage-current relationship from the model. (C) A current-clamp simulation in the model using the same stimulation protocol as in A. (D) A representative voltage-clamp recording from a blowfly photoreceptor in a dissociated ommatidium; series resistance and whole-cell capacitance were compensated, and liquid junction potential of 16 mV subtracted. (E) Deactivating tail currents from the same patch-clamp experiment. (F) Time constants of *I*_KF_ obtained in patch-clamp experiments; activation time constants were measured by fitting the second halves of current onsets with a sum of two exponential equations; deactivation time constants were determined by fitting the initial several hundred milliseconds (usually 200 ms) of the decaying currents with a sum of two exponentials; each point is average of 2 to 6 data points; error bars are s.d.; red trace is a fit with a voltage-dependent relaxation equation. (G) Time constants of the fast (*I*_KF_) and slow (*I*_KS_) components of the voltage-activated K^+^ current obtained under different experimental conditions and in the model; black trace and blue circles: correspondingly, the fit of time constants of *I*_KS_ from the previous study [30] and time constants from our model; red trace from F; black circles are time constants of *I*_KF_ from the previous study [30]; green squares are activation time constants from the model. (H) The total K^+^ current includes *I*_KF_ and *I*_KS_ in proportion 4:1, and also passive leak current.

The blow fly model was generated in a similar way. Fig 3A shows a representative current-clamp recording. Fig 3B shows sustained voltage-current relationships for 20 photoreceptors, with *V*_rest_ subtracted. It can be seen that variability between *C*. *vicina* photoreceptors in *g*_pas_ was much smaller than in the cockroach (Fig 2B). Median *g*_pas_ was 59(50:72) MΩ. The sustained voltage-current relationship from the model is shown in blue in Fig 3B, and the simulated current-clamp is presented in Fig 3C.

To model the blow fly’s K^+^ conductances, two sources of experimental data were used. In the early study, Weckstrom et al. attempted recording K^+^ currents from photoreceptors in vivo and also recorded from photoreceptors in dissociated ommatidia using the patch-clamp method [30]. The properties of K^+^ conductances were reconstructed from single-channel recordings in isolated patches. It was discovered that blow fly photoreceptors express two distinct sustained K^+^ conductances, a predominant fast-activating (*I*_KF_) and a slow-activating (*I*_KS_) ones [30].

In the previously published study [23], we performed whole-cell patch-clamp recordings from photoreceptors in dissociated blow fly ommatidia. A representative example revealing the presence of two voltage-activated K^+^ currents is shown in Fig 3D. For the model, activation constants and proportions of *I*_KF_ and *I*_KS_ had to be determined. This was accomplished using currents elicited by both activation and deactivation protocols (Fig 3D and 3E). Current onsets (activation recordings, Fig 3D) or decays (deactivation recordings, Fig 3E) were fitted with a sum of two exponential equations. However, only some traces had *I*_KF_ and *I*_KS_ that could be separated by this method. For instance, in Fig 3E, *I*_KS_ was found in three most depolarized traces, and its contribution did not exceed 30%. Fig 3F summarizes results of trace fitting for *I*_KF_. Activation and deactivation time constants overlapped in the range from -65 to -35 mV. The voltage-dependence of activation constants was fitted with a voltage-dependent relaxation equation. Because of data scarcity, no similar voltage-dependence could be obtained for *I*_KS_. Fig 3G compares time constants from the two experimental studies and our model.

Similarly to the cockroach model, if the simulated *I*_KF_ had activation time constants consistent with the patch-clamp results, the initial transient in the current-clamp at depolarized potentials would be overly high and wide. Therefore, to properly model the initial part of the voltage response, we had to use *I*_KF_ activation constants from the study by Weckstrom et al. [30], which are much smaller than the patch-clamp values (Fig 3G). *I*_KS_ activation time constants were also adopted from that study. Green squares and blue circles denote activation time constants of the modeled *I*_KF_ and *I*_KS_, respectively (Fig 3G). Fig 3H shows the total K^+^ current used in the model that included *I*_KF_ and *I*_KS_ in proportion 4:1, and a passive leak current. Although at positive membrane potentials the K^+^ current demonstrated slow inactivation (Fig 3D), we did not introduce it to the model because its onset was outside of the physiological voltage response range.

### Visibility of microvillar signals

By using *P*. *americana* and *C*. *vicina* models, we next investigated visibility in the soma of light responses from the microvillus. The models differed in the diameter of microvilli (40 nm in *C*. *vicina* and 100 nm in *P*. *americana* model), size of the soma, and K^+^ conductances. A transient current or voltage response resembling a QB or, generally, an impulse response (IR), can be simulated in NEURON using a point process called alpha-synapse by assigning its reversal potential at 10 mV, the value characterizing experimental LIC. By altering the time constant of the point process, its kinetics and characteristic duration can be regulated. In the following, a point source simulating a transient IR is referred to as an IR source. IR recorded in the current-clamp mode is henceforth referred to as the voltage IR and that in the SEVC mode as the current IR.

We first tested visibility of IR source located in the soma, by changing positions of the source and the recording site along the soma. The maximal IR source conductance (*g*_max,IR_) was 0.5 nS, yielding a voltage IR of about 1 mV. Current IR half-width was 20.8 ms. These parameters describe a typical dark-adapted QB in both modeled species [31]. Regardless of the position of the IR source or the recording site in the soma, voltage response amplitudes were identical. Only when *g*_max,IR_ increased by 100-fold, eliciting voltage IRs of ~35 mV in amplitude, very slight differences in voltage IR kinetics appeared.

**Fig 4:**
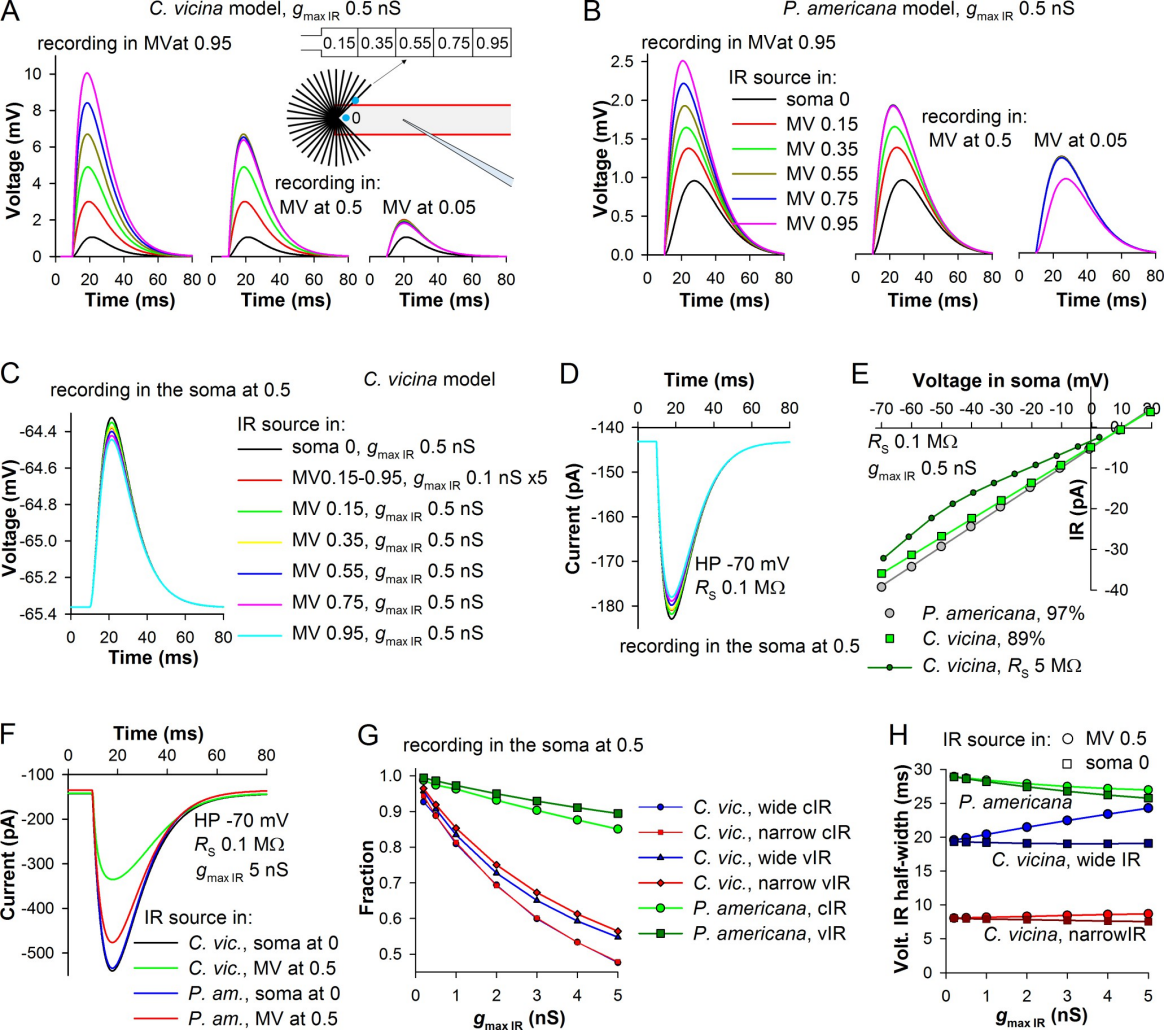
Visibility of impulse responses. (A, B) Voltage IRs in the microvillus (MV) of the blow fly (A) or cockroach (B). IR source was placed in the microvillus or in the soma at 0 (inset in A), and recordings were made at three positions: 0.95 (left sub-panels), 0.5 (center sub-panels), and 0.05 (right sub-panels). Inset in A: Schematic depiction of the model and experiment. (C, D) Visibility of voltage (C) or current (D) IRs from the middle of the soma depending on the position of IR source; the numbers in the legend to C correspond to the positions in the inset to A. (E) Visibility of IR conductance from the soma in SEVC simulations. IR source was situated in the middle of the microvillus. Current IR amplitude was recorded in the soma while clamping the model at different potentials in the range from -70 to 20 mV in 10 mV increments; *R*_s_ was 0.1 MΩ except for one experiment in *C*. *vicina* model as indicated; percentages denote the fractions of maximal IR conductance visible from the soma. (F) Strong attenuation of large current IRs. (G) With increasing conductance of the IR source in the middle of microvillus, fractions of IR conductance (cIR) visible in the soma decreased stronger than the fractions of voltage IR (vIR) amplitudes; voltage IR fraction was measured by dividing the amplitude of the voltage IR originating in the microvillus by that of the voltage IR originating at 0 in the soma, with recording performed in the middle of the soma at *V*_rest_. (H) Changes in half-widths of voltage IRs recorded at *V*_rest_.

In contrast, when both the IR source and recording site were placed in the microvillus, the resulting voltage signal depended strongly on the respective positions. When the recording site was at the distal end of the *C*. *vicina* microvillus (at 0.95), shifting the position of IR source proximally caused a strong progressive decrease in voltage response amplitudes (Fig 4A, left). As the recording site was moved proximally, voltage response amplitudes decreased, with IR sources distally to the recording site eliciting almost identical responses (Fig 4A, center and right). All IR sources located in the microvillus elicited voltage responses larger than that evoked just outside the microvillus in the soma at 0. In the cockroach model, the trend was similar but voltage responses in the microvillus were comparatively small.

All these differences within and between the models can be explained by differences in input resistance along the microvillus. Small, high-resistance microvillus opens at one end into a low-resistance soma, and this creates a gradient of membrane resistance along the microvillus so that the same IR source can elicit different voltage responses depending on the local resistance value. The influence of the soma is smallest at the sealed distal end of the microvillus, and there the IR evoked in the blow fly model was 4 times bigger than that in the cockroach model (Fig 4A and 4B, left panels). However, the difference in the membrane areas, and hence in membrane resistance (*g*_pas_ density was the same), between the blow fly and cockroach microvilli was 2.5, inconsistent with the difference in the voltage response amplitudes. The mismatch disappeared when the microvillus neck diameter in the cockroach model was set to match that in the blow fly model, 20 nm. This increased the amplitude of voltage response that was evoked and measured at the distal end of the cockroach microvillus from 2.5 to 4.05 mV, yielding the 2.5-fold amplitude difference.

Next, we tested how changes in the position of IR source in the microvillus were reflected at the recording site in the middle of the soma (*C*. *vicina* model was used). Fig 4C and 4D compares voltage and current IRs originating from different loci in the microvillus and at position 0 in the soma. The smallest responses corresponded to the IR source at the distal end of the microvillus (Fig 4A, inset, position 0.95) and vice versa. However, overall differences were small. When instead of a single 0.5 nS IR source in the middle of the microvillus we placed five 0.1 nS IR sources along its length, the two responses coincided, indicating that a point source can adequately model activation of distributed light-activated conductance when observed from distance.

Next, we evaluated the visibility of the microvillar IR conductance in the soma. This question is essential for the evaluation of two previously published fly photoreceptor models (see below). By measuring in the soma the amplitude of a current IR from the microvillus in the SEVC mode while changing holding potential in the soma, a current-voltage relationship can be established. Its linear fitting yields a slope factor that equals conductance, which the IR source situated in the microvillus contributes to the somatic conductance. Fig 4E presents three simulations using a small IR source (*g*_max,IR_ 0.5 nS). In the cockroach model, the IR source contributed 97% of its original 0.5 nS. In the blow fly model characterized by narrow microvilli, the contribution decreased to 89%. However, when in *C*. *vicina* model instead of 0.1 MΩ series resistance we used 5 MΩ, the voltage-dependence of IR amplitudes has become non-linear.

Although small QB-like depolarizations added almost all of their associated conductance to the somatic conductance, it was necessary to conduct similar tests for the current responses elicited by higher *g*_max,IR_, and also to study how properties of voltage responses evoked in the microvillus change with the increasing *g*_max,IR_. In the blow fly model, when *g*_max,IR_ increased by 10-fold, the amplitude of the current IR originating in the microvillus and recorded in the middle of the soma decreased by 2-fold compared to the IR originating in the soma at 0 (Fig 4F). In the cockroach model, the decrease was comparatively small. The decrease is caused by incomplete clamping of the microvillus so that the residual voltage response of the voltage-clamped microvillus reached tens of millivolts when large *g*_max,IR_ were used. Because the driving force of the IR source decreases with depolarization, the incomplete clamping causes progressive curtailing of the depolarizing IR and its diminishment at the recording site in the middle of the soma.

These simulations are summarized in Fig 4G and 4H. In the blow fly model, the fraction of IR source conductance lost with increasing *g*_max,IR_ was much larger than in the cockroach model (Fig 4G). In addition to the wide IR generated by an IR source with a time constant of 8 ms (IR half-width of 20.8 ms), we also investigated changes in the visibility of an IR source with a time constant of 2 ms, which elicited a current IR with a half-width of 5.2 ms. The narrow IR approximates the strongly light-adapted IRs of blow fly photoreceptors [31]. However, there was no difference in the visibility of wide and narrow IRs (Fig 4G). Because of comparatively small amplitudes of voltage IRs evoked by the same IR sources (Fig 4A and 4B), visibility of microvillar conductance in the soma of the cockroach model was substantially higher (Fig 4G).

Next, we studied changes in visibility of voltage IRs. We divided the amplitude of voltage IR from the microvillus as recorded in the middle of the soma by the amplitude of IR evoked in the soma at 0. It was found that visibility of voltage IRs in all instances was better than that of conductances (Fig 4G). Changes in voltage IR properties were not limited to amplitude attenuation. Fig 4F demonstrates that the characteristic duration of voltage IRs evoked in the microvillus tended to increase with the growing *g*_max,IR_, especially in the blow fly model.

### Modulation of impulse response by voltage-activated and light-induced conductances

Previous studies described many interactions between the depolarizing light-induced and repolarizing voltage-activated K^+^ conductances in rhabdomeric photoreceptors. Although the majority of studies focused on the roles of specific K^+^ conductances in shaping the voltage response generated by light-induced current (LIC) [12, 17, 18, 32, 33], there is also evidence of interactions between the delayed rectifier K^+^ currents and LIC that can cause either amplification (in *D*. *melanogaster* [34]) or suppression (in *P*. *americana* [19]) of the delayed rectifiers.

**Fig 5:**
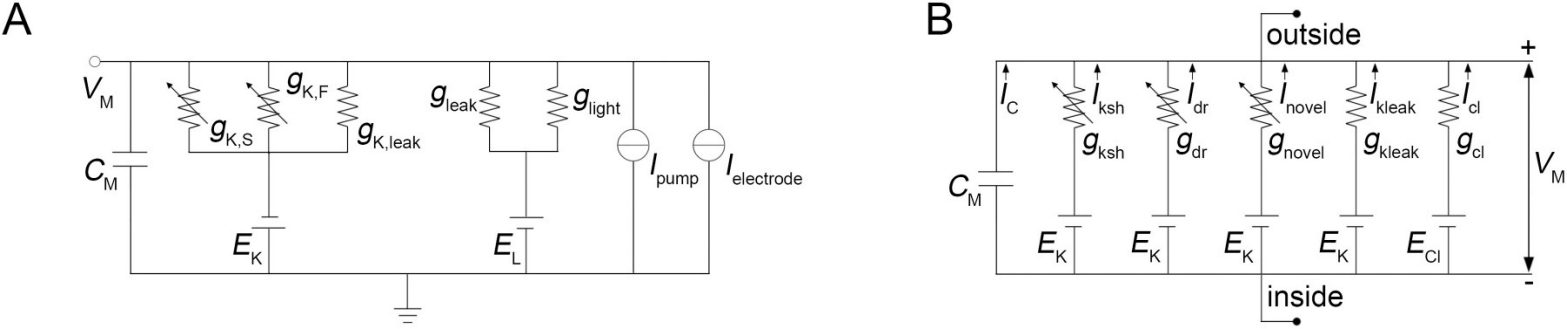
Two electrical models of rhabdomeric photoreceptors. (A) Model by Heras, Laughlin and Niven [12]. (B) Schematic of the somatic module of the model by Song and Juusola [14].

Although fly photoreceptors are modeled electrophysiologically for over two decades, there is still no consensus regarding the basic structure of the model. Fig 5 compares two electrical circuit models of the photoreceptor membrane. In Fig 5A, a model by Heras, Laughlin and Niven [12] incorporates *C*_m_, two current sources (sodium-potassium pump and electrode), the voltage-activated and leak K^+^ conductances (*g*_K,F_, *g*_K,S_ and *g*_K,leak_, respectively) with their K^+^ reversal potential *E*_K_, LIC conductance (*g*_light_) and a depolarizing leak conductance (*g*_leak_) with their reversal potential *E*_L_. The circuit in Fig 5B represents the photoreceptor body part of the model by Song and Juusola [14]. It consists of *C*_m_, three voltage-activated (*g*_ksh_, *g*_dr_ and *g*_novel_) and a leak (*g*_leak_) K^+^ conductances with their K^+^ reversal potential *E*_K_, and a Cl^-^ conductance with its reversal potential *E*_Cl_. Importantly, the latter model does not include LIC conductance. Instead, “the voltage responses are generated by injecting the macroscopic LIC to a Hodgkin–Huxley model of the photoreceptor cell body membrane” [14]. It follows that LIC is used exclusively as a current source, similarly to the electrode current in the current-clamp mode, with LIC electromotive force regulated by voltage feedback.

The differences between two models are significant and need to be tested. The principal question is how LIC conductance per se can affect responses to light produced by the phototransduction cascade. The simplest testing approach would be to compare properties of models depolarized either by a current injection or by LIC. The first scenario would correspond to the Song-Juusola model and the second to the Heras-Laughlin-Niven model. The state of the system can be probed by injecting a depolarizing current simulating a QB, which is an irreducibly short electrical response of the photoreceptor. The resulting voltage IR would encode all temporal information about the current state of the system. The amplitude spectrum of the impulse response gives the system’s gain function, from which the corner frequency of signal transfer can be obtained.

**Fig 6:**
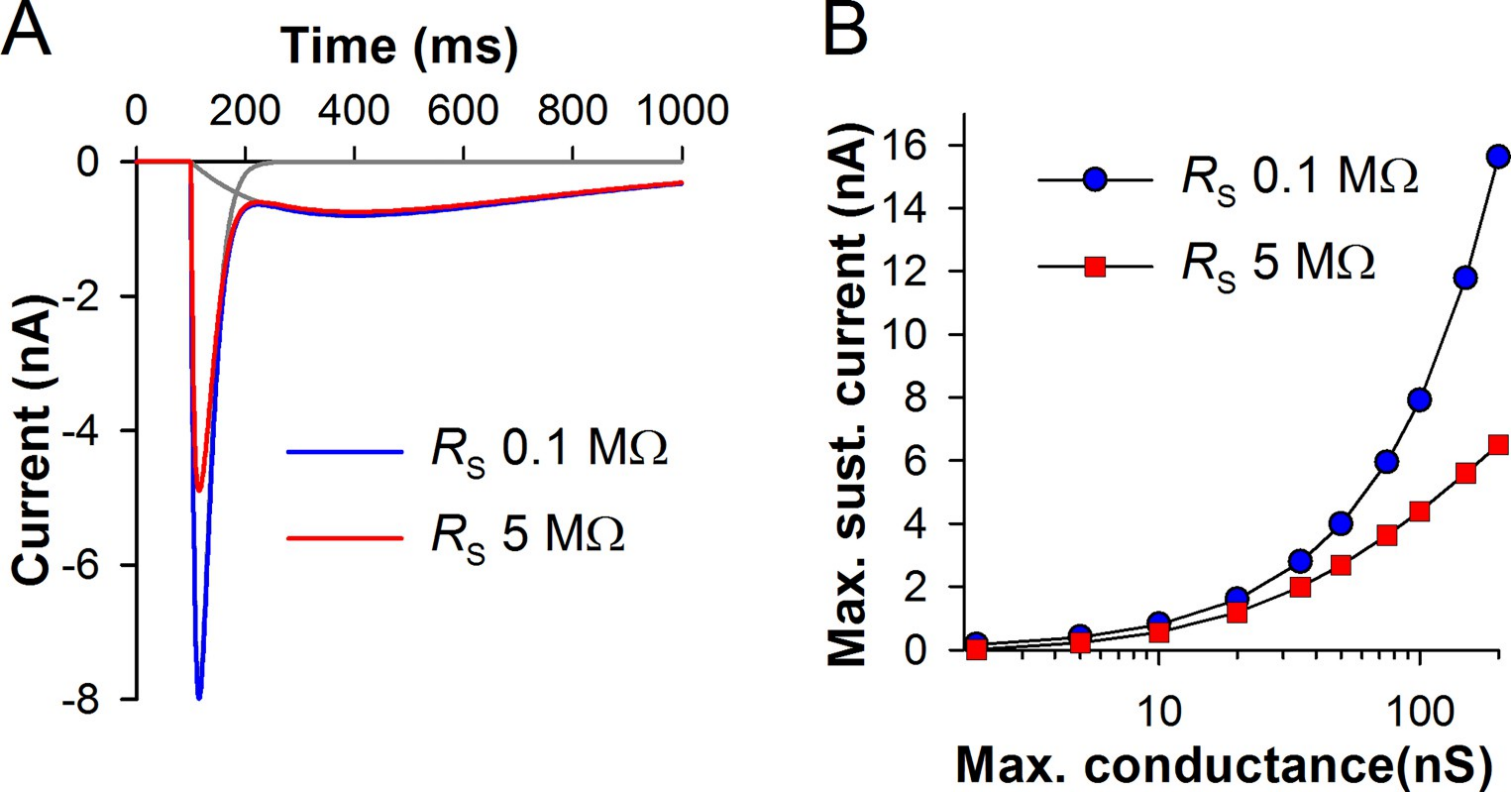
Modeling LIC in SEVC. (A) By combining two inward current sources (grey), a current response resembling a typical light-induced current (blue) can be obtained; red trace: a simulated LIC recorded using an electrode with a realistic series resistance. (B) Effects of the series resistance on the amplitude of the sustained component (at ~400 ms in A).

By modifying kinetics of an IR source, it is possible to simulate sustained depolarizing current. Fig 6A shows that by combining two IR sources, a current response waveform resembling LIC as it is recorded from photoreceptors in patch-clamp experiments can be obtained. The transient component appears when a dark-adapted photoreceptor is stimulated by comparatively bright light. The sustained component can be maintained for minutes, depolarizing photoreceptors up to about -20 mV in the brightest light. To simulate sustained LIC we employed an IR source with a time constant of 500 ms, placing it in the soma (henceforth, LIC source). The amplitude of the sustained LIC in Fig 6A corresponds to a typical LIC in bright light in cockroach photoreceptors [35]. We also evaluated the effect of series resistance on LIC. Although the transient LIC was strongly suppressed, sustained LIC was almost unchanged. Fig 6B shows that the measurement error for the sustained LIC becomes substantial at such large LIC amplitudes that are almost never registered in patch-clamp experiments.

**Fig 7:**
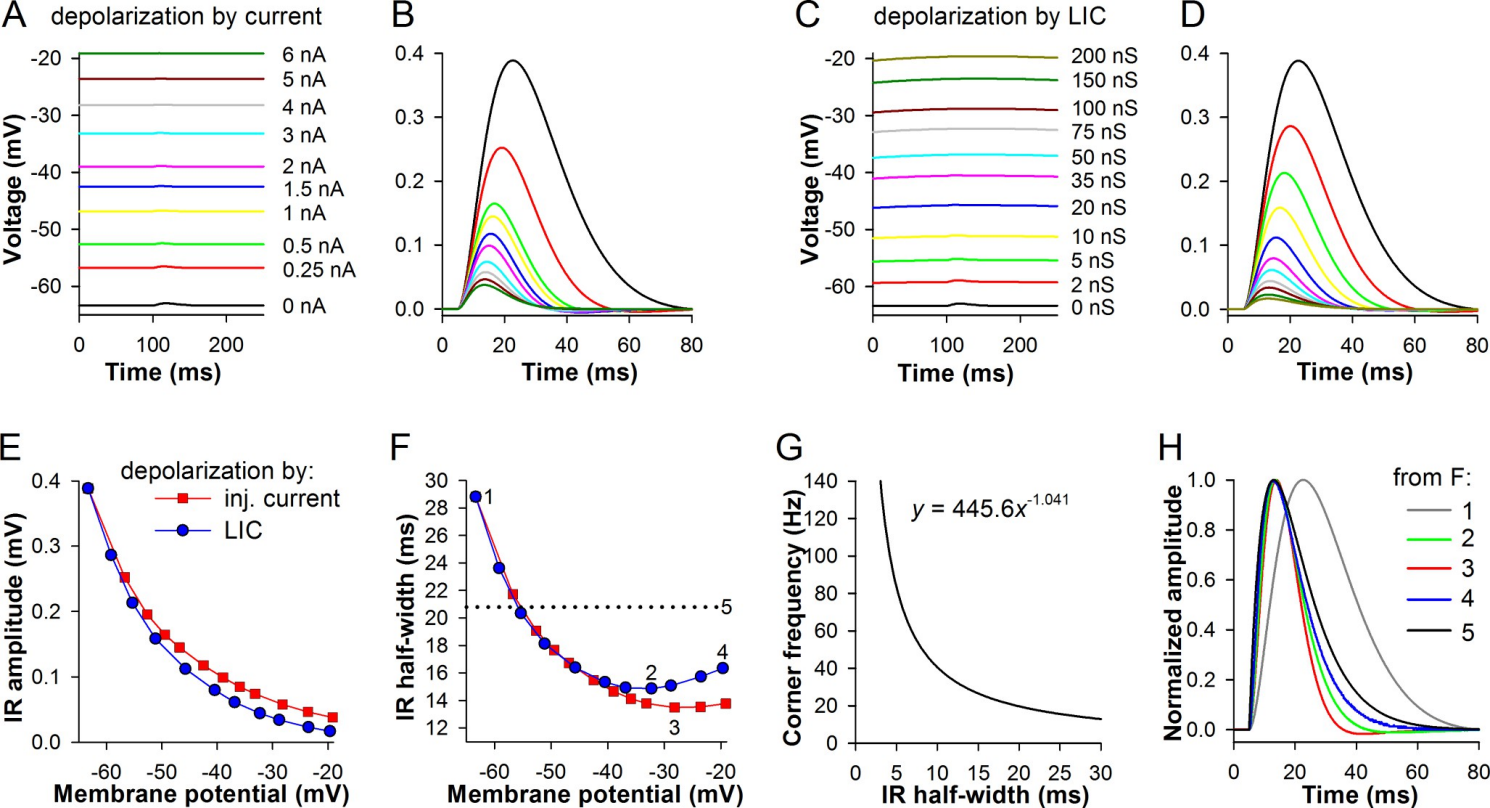
Transformations of impulse responses by depolarization in *P*. *americana* model. (A) The model cell was depolarized by current pulses (amplitudes to the right). During each pulse, a small (*g*_max,IR_ 0.2 nS) current stimulus was applied at 100 ms. (B) The superimposed voltage IRs isolated from A (color coding is the same for A-D). (C) Depolarization by sustained LIC; the time constant of the IR source was 500 ms and *g*_max,IR_ increased as indicated by the values to the right. (D) Superimposed voltage IRs from C. (E) Dependencies of voltage IR amplitudes on membrane potential. (F) Dependencies of voltage IR half-widths on membrane potential; the horizontal dotted line denotes the half-width of the current stimulus (20.8 ms). (G) The empirical function to convert IR half-width to corner frequency; the curve was generated using a series of lognormal distribution-shaped waveforms with different half-widths that simulated IRs; corner frequencies were obtained from the amplitude spectra and plotted against the half-widths of the waveforms. (H) Normalized IRs corresponding to numbers in D.

Fig 7 describes the experiment in the cockroach model. First, the model was depolarized by current injections, with the pulse magnitudes shown to the right (Fig 7A). The state of the system was probed using an IR source with a *g*_max,IR_ 0.2 nS and time constant of 8 ms (at ~100 ms in Fig 7A). The half-width of the probing current was 20.8 ms, which is typical for the cockroach QBs and voltage IRs in vivo [29]. Voltage IRs isolated from the traces are shown in Fig 7B. Next, the model was depolarized by a sustained LIC (Fig 7C). Because this did not produce a flat voltage background, to properly isolate the IRs we additionally generated an identical set of traces without IRs, and then subtracted the traces. When IR amplitudes were plotted against the corresponding membrane potentials, significant differences have emerged (Fig 7E). Because LIC contributes additional conductance, the amplitudes of voltage IRs decreased stronger during depolarization by LIC than during depolarization by injected current.

Changes in IR half-widths were non-trivial (Fig 7F). Firstly, the voltage IR half-widths decreased sharply with depolarization, at about -55 mV becoming smaller than the half-width of the IR in the voltage-clamp mode (Fig 7F, horizontal dotted line). This is a crucial novel finding suggesting that voltage-activated K^+^ channels can sharpen the IR generated by phototransduction, actively increasing the photoreceptor signaling bandwidth. Corner frequencies (*f*_c_) of signal transfer can be estimated from the IR half-widths using the empirical conversion function shown in Fig 7G. The probing current had a half-width of 20.8 ms that corresponded to an *f*_c_ of 18.9 Hz (Fig 7F, #5). At *V*_rest_, voltage IR was amplified and low-pass filtered by the membrane, with the half-width of 28.8 corresponding to an *f*_c_ of 13.5 Hz (Fig 7F, #1).

Secondly, the half-width of voltage IRs from the depolarization by current injection experiment reached a minimum of 13.5 ms at -28 mV (Fig 7F, #3), giving an *f*_c_ of 29.7 Hz. At more depolarized voltages, the IRs had slightly widened. Thirdly, at voltages above -40 mV, the half-widths of voltage IRs from the depolarization by LIC experiment were larger than those from the current injection experiment. The half-width minimum of 14.9 ms at -32.3 mV corresponds to an *f*_c_ of 26.8 Hz (Fig 7F, #2). At more depolarized voltages the half-widths increased, reaching 16.3 ms at -19.6 mV, which corresponded to an *f*_c_ of 24.3 Hz (Fig 7F, #4). Fig 7H shows normalized IRs that were numbered in Fig 7F. Voltage IR #1 is the widest and has the slowest onset, a sign of low-pass filtering. Onsets of other voltage IRs almost coincided with that of the current IR (#5, black), indicating a negligible low-pass filtering. Comparison of IR decays clearly shows the effect of repolarizing K^+^ current. Decays of red and green traces (#3 and 2, respectively) were characterized by hyperpolarizations absent in other traces.

**Fig 8:**
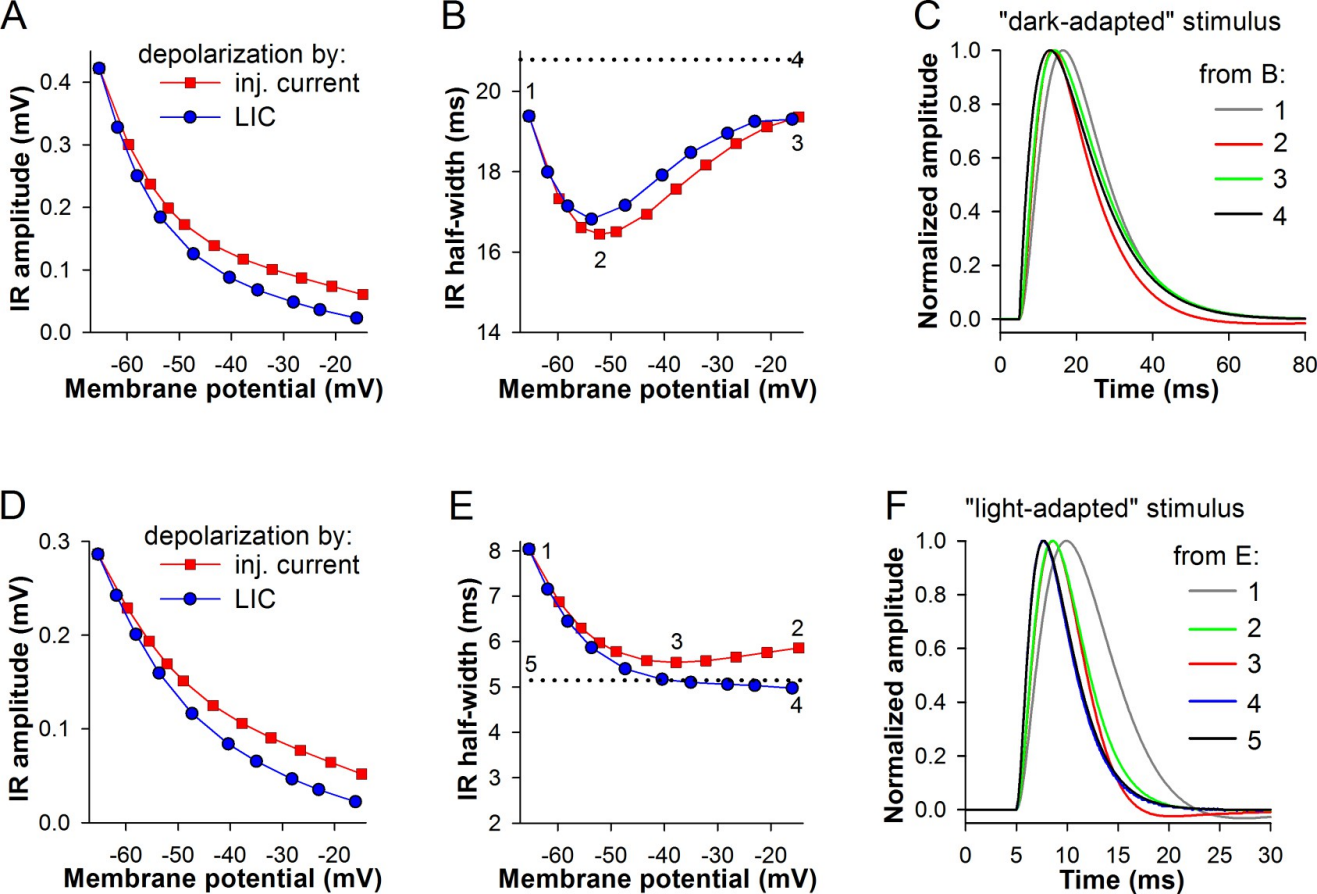
Transformation of impulse responses by depolarization in *C*. *vicina* model. (A-C) Changes in voltage IR amplitudes (A) or durations (B) during depolarization by current injections or background LIC; stimulations were performed using the same current or LIC pulses as in Fig 7A and 7C; a wide current stimulus (half-width 20.8 ms) approximating a dark-adapted QB was used. (C) Normalized IRs from B. (D-F) The same as in A-C, except that a narrow current stimulus (half-width 5.2 ms) approximating a light-adapted QB was used.

Similar experiments conducted in the blow model yielded only partly consistent results. Because, unlike in the cockroach, light adaptation of the phototransduction cascade in the flies in addition to amplitude attenuation shortens the QBs [31], we used two IR sources with time constants of 8 and 2 ms, i.e. the same as in Fig 4G and 4H. The former simulated a dark-adapted IR and the latter a light-adapted one. Whereas IR amplitudes decreased in a similar fashion with depolarization (Fig 8A and 8D), voltage-dependencies of IR half-widths were dissimilar. In the simulations employing a wide IR, the half-widths of IRs at *V*_rest_ were smaller than that of the probing current, indicating that low-pass filtering in this model did not restrict signaling bandwidth even at *V*_rest_. The IRs were characterized by sharp half-width minima at about -53 mV. The minimal half-widths exceeded those in the cockroach simulations, rebounding back toward the *V*_rest_ value with further depolarization (Fig 8B). These results suggest that the fast K^+^ conductance used in the blow fly model shortens wide stimuli relatively inefficiently. Comparison of the shortest IRs from Figs 7H and 8C (red traces) reveals a comparatively strong effect of the cockroach K^+^ current on the decay of the IR.

When the blow fly model was probed with a narrow current stimulus, the resulting voltage-dependencies of IR half-widths had a minimum only in the current injection experiment (Fig 8E). In contrast to the simulations with wide testing stimuli, depolarization by LIC sharpened the voltage IRs to a larger extent than depolarization by current pulses. At *V*_rest_, voltage IRs were strongly low-pass filtered (Fig 8F, dark gray trace). The filtering relaxed only in the depolarization by LIC experiment, at membrane potentials above -40 mV, as indicated by the fully coinciding voltage IR #4 (blue trace) and the current stimulus (#5, black).

### Experiments support peaking of corner frequency

Our modeling results suggested that: (1) voltage-activated K^+^ conductances can sharpen voltage IRs beyond the limit set by phototransduction, significantly increasing corner frequency of signal transfer; and (2) the effect is voltage-dependent, so that a minimum of IR half-width can be observed at a certain depolarization level.

Is there experimental support for the findings? On one hand, data allowing direct comparison of current and voltage IRs or prolonged responses to contrast-modulated stimuli can in practice be obtained only in patch-clamp recordings from photoreceptors in dissociated ommatidia. However, previous studies indicate that functioning of photoreceptors in the ex-vivo experiments is severely compromised. In the fruit fly, patch-clamped photoreceptors quickly undergo run-down [36]. In the cockroach, photoreceptors in such experiments can withstand prolonged stimulation but their IRs are much wider than in vivo, i.e. have lower corner frequencies [29]. On the other hand, the voltage-dependence of corner frequencies can be studied in the conventional intracellular recording experiments. Because light adaptation of the phototransduction cascade in flies involves shortening of the QB, proper experimental methodology to study voltage-dependence of corner frequencies should include repetitive stimulation at one light level while changing membrane potential by injecting depolarizing current. However, because IR shortening during light adaptation is progressive [31], observing a corner frequency maximum during conventional experiments that involve incrementing light stimulation would also lend support to our in-silico findings.

**Fig 9:**
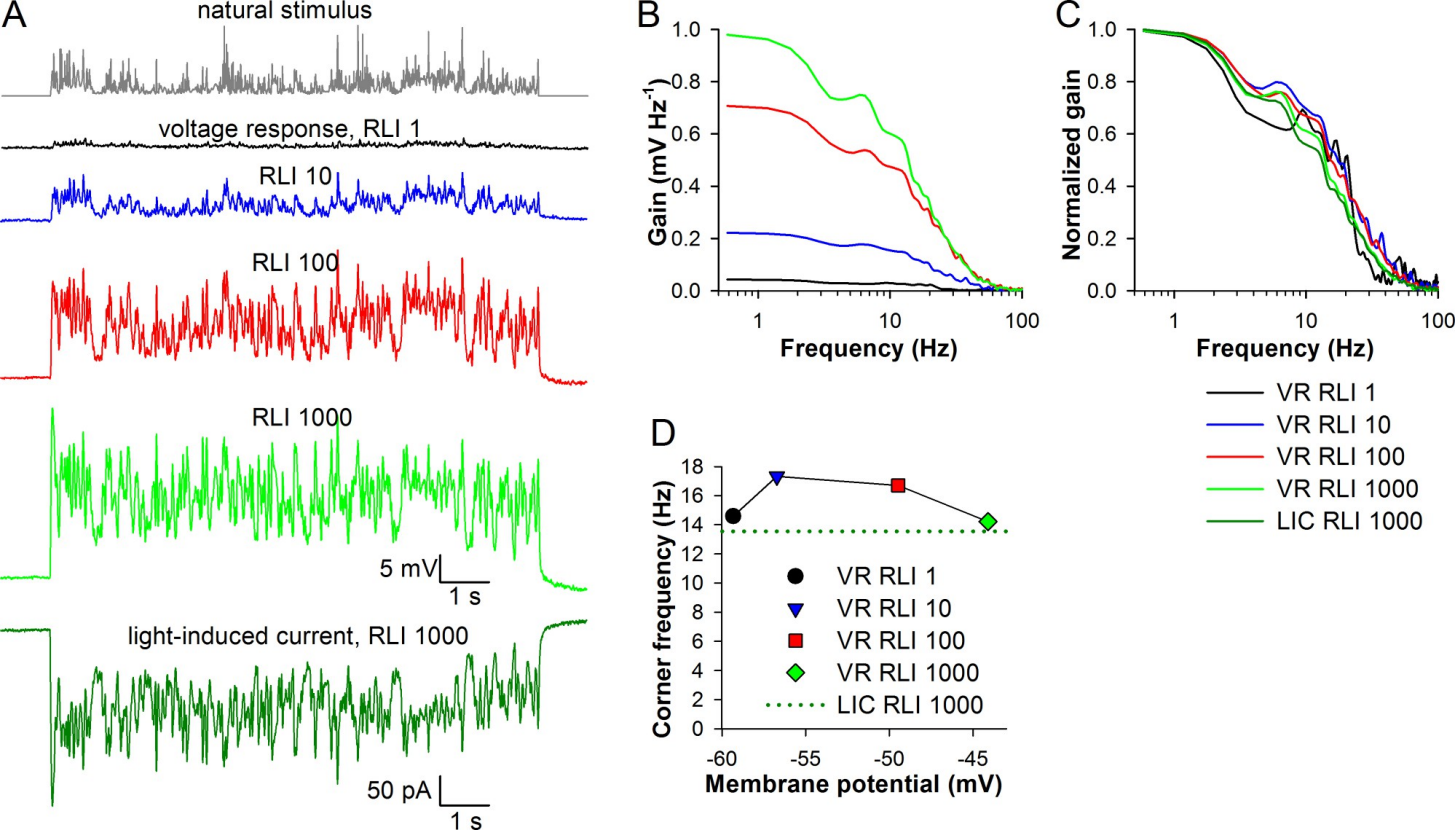
Blow fly photoreceptor responses to natural stimulation in vitro. (A) Voltage responses of a photoreceptor from dissociated ommatidium to a 10-s natural stimulus (above) at four relative light intensities (RLI) as indicated; the lowest trace is a current response recorded at a holding potential of -84 mV. (B) Signal gain functions for voltage traces from A. (C) Normalized gain functions of the voltage and current responses from A. (D) Dependence of corner frequencies on the average membrane potential of the voltage responses; the dotted dark green trace denotes the corner frequency of the LIC trace.

We obtained patch-clamp recordings from a blow fly photoreceptor (Fig 9). Photoreceptor was stimulated by a 10-s natural light contrast (Fig 9A, top trace). Voltage responses were recorded at four light intensities (relative light intensity, RLI) in 10-fold increments. LIC response was recorded only at the brightest intensity (Fig 9A, bottom). Fig 9B shows signal gain functions for voltage responses, and Fig 9C normalized gain functions for both the voltage and LIC responses. The corner frequency, defined as the frequency at which signal gain decreased by 50%, was obtained by fitting the signal gain functions in the range 1–200 Hz with a Hill equation. Fig 9D shows voltage-dependence of the corner frequencies, with voltages denoting the mean sustained voltage response amplitudes. These results indicate that all voltage response corner frequencies exceed that of the LIC response, and that corner frequencies reach maximum at -57 mV. The results are consistent with our modeling conclusions.

**Fig 10:**
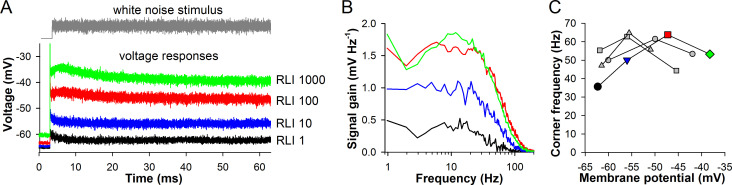
Changes in the corner frequencies in the hover fly. (A) Voltage responses of a hover fly photoreceptor in vivo to a 60-s white-noise modulated stimulus at four intensities as indicated. (B) Signal gain functions for traces from A. (C) Dependencies of gain corner frequencies on the average sustained membrane potential for four cells; the colored trace denotes the photoreceptor from A.

Fig 10A shows intracellular recordings from a hover fly (*Volucella pellucens*) photoreceptor. The cell was stimulated with a 60-s white-noise modulated light at four intensities in 10-fold increments. The corresponding signal gain functions are shown in Fig 10B. Voltage-dependencies of corner frequencies for this and three other photoreceptors are presented in Fig 10C. In all experiments, a clear maximum of corner frequencies was observed.

## Discussion

In this study, we first investigated the visibility of the rhabdomeric membrane from the soma, then the visibility of signals originating in the microvillus, then the contribution of the light-induced conductance to the total membrane conductance, and, finally, the interactions between voltage-activated K^+^ and light-induced conductances in their modulation of voltage responses to light. The novel computational results presented here eliminate uncertainties about the influence of the peculiar rhabdomeric topology on the photoreceptor electrical properties. Most importantly, we for the first time show that voltage-activated K^+^ conductances can increase light response’s bandwidth beyond that set by the phototransduction.

### Photoreceptor size and functional parameters

In the previous studies, we established dependencies of several electrophysiological properties and signal processing parameters on *C*_m_. Photoreceptor sensitivity to light correlates strongly positively with *C*_m_ in *P*. *americana* [4, 28, 35], *Panchlora nivea* [37], *Corixa punctata* [38], *Notonecta glauca* [39], *Gerris lacustris* [9], and *Carausius morosus* [8], presumably because sensitivity of a given cell is proportional to the area of the light-sensitive rhabdomeric membrane, which is reflected in *C*_m_. For the same reason, LIC amplitude correlates positively with *C*_m_ in *C*. *morosus*, *G*. *lacustris*, *C*. *punctata* and *P*. *americana* [8, 9, 35, 38]. *I*_DR_ amplitude correlates positively with *C*_m_ in *C*. *morosus*, *G*. *lacustris*, *N*. *glauca*, and *P*. *nivea* [8, 9, 37, 39]. Voltage-activated K^+^ channels are expressed in the somatic membrane, so the positive but not very strong correlations probably account for the variation in the size of the light-insensitive membrane.

The maximal information rate correlates positively with *C*_m_ in *C*. *morosus*, *G*. *lacustris*, *N*. *glauca* and *C*. *punctata* [8, 9, 38, 39]. The maximal corner frequency correlates positively with *C*_m_ in *G*. *lacustris* and *C*. *punctata* [9, 38]. Information rate is determined by the integral of signal-to-noise ratio (SNR) over the signaling bandwidth. While SNR is expected to increase with the number of microvilli, which are the receptor sampling units, the positive correlation between *C*_m_ and corner frequency is more difficult to explain. One possibility could be that cells with large rhabdoms have disproportionally big *I*_DR_ channel densities. However, testing this hypothesis requires more data than currently available.

Because *C*_m_ depends on both the light-sensitive and insensitive parts of the photoreceptor membrane, with proportions of the two membrane fractions differing between species [2], it was necessary to establish to what extent *C*_m_ measurement in the soma can account for the light-sensitive membrane of the microvilli. Here, our experiments in the model showed that even the ultra-narrow microvilli of the blow fly contribute their membrane fully to the whole-cell *C*_m_, even after their membrane resistance is lowered by 100-fold. Isopotentiality of the rhabdomere is lost only when the narrow microvillus is extended to the lengths never found in nature (~10 μm). Therefore, our results indicate that *C*_m_ measurements from the soma probably faithfully represent the entire photoreceptor membrane. Interestingly, the microvillus neck did not have any significant effect on *C*_m_ estimates.

There is currently no explanation for the variability in the dimensions of the microvilli between species. Assuming the uniform density of rhodopsin, elongation of the microvillus within the rhabdom can facilitate light capture, and so the nocturnal and crepuscular species indeed tend to have longer microvilli than the purely diurnal ones [2, 40].

### Visibility of microvillar signals and light-induced conductance

Light-activated signals originate in the microvilli. Compared to the soma, microvillus is tiny and its membrane resistance at rest is proportionally larger. The elementary IR was modeled by placing the IR source in the microvillus and adjusting its parameters so as to evoke a small quantum bump-like event in the soma. The source triggering 1 mV voltage IR in the soma can generate an order of magnitude larger response in the narrow blow fly microvillus, depending on the positions of the IR source and the recording site (Fig 4A). The variability is due to the large low-resistance soma having a strong effect on input resistance in the microvillus, which thus changes along the microvillus. Interestingly, decreasing the diameter of the microvillus neck can increase the amplitude of voltage response in the microvillus. This can have physiological consequences because increased depolarization reduces driving force for LIC, attenuating influx of both Na^+^ and Ca^2+^ and decreasing the visibility of the microvillar signal in the soma (see below).

When voltage or current response was recorded from the middle of the soma, moving a small conductance IR source along the microvillus or even placing it in the soma outside of the microvillus had little effect on the amplitude or kinetics of the recorded signal (Fig 4C and 4D). Although the signal was the largest when the IR source was situated in the soma and the smallest when it was at the distal end of the microvillus, the difference in amplitude in the blow fly model was only ~11%.

Estimations of conductance indicated that for the IR source with *g*_max,IR_ of 0.5 nS almost all conductance from the cockroach microvillus (97%) and a large fraction of conductance from the blow fly microvillus (89%) was added to the somatic conductance (Fig 4E). However, the fraction of transferred conductance depends strongly on the maximal conductance of IR source, i.e. on the amplitude of the voltage or current IR generated in the microvillus. When *g*_max,IR_ was lowered from 0.5 to 0.2 nS, the visibility of microvillar conductance in the blow fly model increased from 89 to 93% (Fig 4G). When *g*_max,IR_ was instead increased, the visibility decreased progressively, to 48% for *g*_max,IR_ of 5 nS in the blow fly model (Fig 4F and 4G). In the cockroach model, the corresponding decrease was much smaller, from 99% for *g*_max,IR_ of 0.2 nS to 85% for *g*_max,IR_ of 5 nS. Similar albeit slightly less prominent trends were observed in the current-clamp simulations for voltage IRs (Fig 4G). The cause of visibility reduction is the same for both current and voltage IRs–a decrease in the driving force for LIC in the microvillus. In the SEVC simulation, voltage clamping error manifests in the form of residual voltage transient in the microvillus, the amplitude of which increases to tens of millivolts for large IR source conductances. This reduces the driving force and attenuates IR current registered in the soma. In the current-clamp simulations, the driving force decreased much stronger during large voltage IRs in the microvillus than during the relatively small reference voltage IR in the soma at 0. Likewise, attenuation of conductance in the cockroach model was much smaller than in the blow fly model because of the relatively small microvillus input resistance. This rendered the voltage IR in the cockroach microvillus smaller and its driving current less affected by the depolarization-induced decrease in the driving force than in the blow fly microvillus.

Therefore, our results indicated that small IRs, originating in the microvillus and corresponding magnitude- and kinetics-wise to the QBs of real rhabdomeric photoreceptors [31], are minimally attenuated at the recording site in the soma, and their conductance is added nearly completely to the total conductance. Our results thus validate the model by Heras et al. [12] that incorporates light-induced conductance alongside other ionic conductances and current sources (Fig 5A). Moreover, when photoreceptor light adapts its QBs diminish, and the fraction of their conductance transferred to the soma can increase further.

#### Interaction between the light-induced and voltage-activated K^+^ conductances

We studied interaction between the light-induced and voltage-activated K^+^ conductances by deploying a simple but previously unused methodology. The electrophysiological state of the model was probed under different sustained response conditions with an inward current stimulus that kinetically approximated a QB. The perturbations produced voltage IRs, which encode all essential information about the momentary state of the system.

We made several unexpected findings. Firstly, sustained voltage-activated K^+^ conductances can conditionally shorten voltage IRs, expanding the photoreceptor signaling bandwidth beyond that set by phototransduction as approximated by the testing stimulus. Secondly, voltage-dependencies of changes in the characteristic duration of voltage IRs had minima within the physiological voltage response range, depending on the kinetics of the voltage-activated K^+^ conductance, the presence or absence of the background light-induced conductance, and the kinetics of the probing current. Thirdly, background LIC can exert opposing effects on the voltage IRs, depending on the parameters of *I*_DR_ and the current stimulus.

Functions of sustained voltage-activated K^+^ conductances in rhabdomeric photoreceptors include: countering depolarization by LIC; lowering membrane resistance and through this the gain of light responses, which also decreases the membrane time constant and relaxes the low-pass filtering; fast-activating *I*_DR_ in the blow fly photoreceptors produces shunt peaking, increasing the gain-bandwidth product of the membrane; and, by accelerating decay of light responses, *I*_DR_ can selectively suppress lower frequencies of the signal gain function [12, 17–19].

The mechanism of IR narrowing by *I*_DR_ can be discerned by comparing normalized IRs in Figs 7H and 8C, 8F. Superposition of the black and red traces in Fig 7H reveals that *I*_DR_ disproportionally opposes the inward current during its decay phase, causing a visible hyperpolarization. This effect was diminished during depolarization by LIC (Fig 7H, green and blue traces). In the simulation in the blow fly model that involved a wide “dark-adapted” current stimulus, the effect of *I*_DR_ at its maximum (Fig 8C, red trace) was smaller and lacked the after-hyperpolarization, decreasing the voltage IR half-width to a smaller extent than in the cockroach model.

The main difference between the models in these simulations was about three-fold slower activation rates of *I*_DR_ in the cockroach (Figs 2G and 3G). Consequently, when the current stimulus depolarized the membrane, cockroach’s *I*_DR_ activated with a greater delay than the blow fly’s *I*_DR_. The observed minima of IR half-widths thus occur at the voltages where the activating *I*_DR_s most strongly counteract the depolarizing stimulus current specifically during its decay phase, or, in other words, where *I*_DR_ activation is optimally delayed. The kinetics of the probing current stimulus is not voltage-dependent but that of *I*_DR_ activation is, hence the observed IR half-width minimum. At more negative potentials, *I*_DR_ activates too slowly and its effect on the IR decay is small. At more depolarized potentials *I*_DR_ activates already during the IR onset, suppressing both its peak and decay, without a strong disproportional effect on the latter.

In the blow fly, the fast (main) component of *I*_DR_ is activated already at *V*_rest_, attenuating low-pass filtering by the membrane. This manifests in the 10-ms voltage IR half-width difference between the models (Figs 7F and 8B). Blow fly’s *I*_DR_ activation time constants at potentials above rest are so small (Fig 3G) that the channels open already at the IR onset and cannot selectively oppose stimulus current during its decay. It follows then that the effect of *I*_DR_ on IR decay should be more pronounced when a stimulus simulating the narrow “light-adapted” quantum bump is used. Indeed, after-hyperpolarization reappeared in the red trace of Fig 8F, which was the shortest voltage IR in the depolarization by current injection experiment. However, in this simulation, because of very high corner frequency associated with the narrow current stimulus (81 Hz), the membrane impedance low-pass filtered the IRs in the absence of background LIC (Fig 8E).

Why did background light-induced conductance decrease the corner frequency in some situations but increase in others (Figs 7F, 8B and 8E)? Essentially, background LIC behaves like a passive inward conductance, which can attenuate low-pass filtering but cannot otherwise modify the shape of voltage IR. This membrane impedance-lowering effect is beneficial when membrane is too slow to accommodate fast transduced currents without distortion, which explains the situation described in Fig 8E. However, when membrane is driven by a wide current stimulus characterized by a narrower bandwidth than that of the membrane, adding a background light-induced conductance has a deleterious effect on the bandwidth of the resulting voltage IR. We hypothesize that this decreases membrane time constant and thus changes the timing of interaction between the *I*_DR_ and the testing stimulus.

In flies, phototransduction cascade adapts to light by decreasing the amplitude, latency and duration of QBs [31]. Shortening of QB latency is accompanied by a decrease in the spread of latencies, which, together with the narrowing of quantum bumps, underlies the shortening of multiphoton impulse response [41, 42]. Since light adaptation is caused by LIC and associated with membrane depolarization, i.e. pseudo voltage-dependent, a question arises about possible matching of *I*_DR_ activation kinetics to the shortening of IRs. Natural selection-driven co-adjustment of these two independent voltage-dependencies could expand the signaling bandwidth at no additional metabolic cost because channel gating is the result of voltage-driven allostery encoded in the protein’s primary sequence.

We examined available experimental data in the blow and hover flies and found support for our main findings in the model (Figs 9 and 10). The patch-clamp recordings in the blow fly demonstrated that: (1) corner frequencies of voltage responses at all intensities were larger than the corner frequency of the current response of the light-adapted photoreceptor, and (2) had a clear maximum (Fig 9D). The first observation was unexpected because blow fly photoreceptors quickly light adapt in vivo and corner frequency of the LIC response at RLI 1000 should be relatively high. However, in the previous study that compared functioning of cockroach photoreceptors in the intracellular and patch-clamp experiments we established that corner frequencies decreased strongly in the latter setting [29]. The same unidentified factor could diminish performance of the blow fly photoreceptors as well.

Although intracellular recording experiments could not provide contrast-modulated LIC responses, maxima of voltage-dependencies for corner frequencies were found in all hover fly photoreceptors (Fig 10). These results are consistent with our modeling conclusions.

### Comparison of two models

We compared two electrical models of fly photoreceptors used in previous studies [12]. The main difference between the models was the absence of light-induced conductance in the Song-Juusola model [14]. The error introduced by replacing light-induced conductance with a depolarizing current source increases the signal gain and parametrically modifies the characteristic duration of the IR and thus the signaling bandwidth. Voltage-dependence of bandwidth modification is determined by the conductance and activation kinetics of *I*_DR_, the kinetics of phototransduction-generated current, the magnitude of LIC, and possibly membrane capacitance since it is a multiplier in the membrane time constant. While disregarding the light-induced conductance may have no bandwidth consequences for depolarizations of up to 20 mV (Fig 7F), the error in gain accumulates progressively with depolarization (Figs 7E, 8A and 8D).

### Response bandwidth modulation in other systems

In the sensory systems, external inputs at the receptor periphery are transduced into ionic currents. To reduce the costs of signal processing and downstream transmission, the receptor currents have the slowest kinetics and thus the narrowest bandwidth that still allows the receptor to carry out its function [43]. Likewise, the postsynaptic potentials evoked at the postsynaptic terminals–the inputs of neurons–vary in kinetics depending on the type of the receptor channels. For instance, voltage responses of the high-affinity glutamate NMDA receptors are slow and prolonged, transferring only low-frequency signals. In contrast, the low-affinity AMPA receptors mediate extremely fast excitatory postsynaptic potentials (EPSP) that kinetically resemble the action potentials [44]. When the cell membrane transforms the receptor current into a voltage transient, any increase in the characteristic duration caused by low-pass filtering means a loss of useful bandwidth. Generally, receptors and neurons deal with the problem by matching the filtering properties of the main components of the membrane’s electrical circuit [17, 45]. To minimize the low-pass filtering, the membrane time constant should not exceed the time constant for the onset of the transduction current. While this can be accomplished by increasing the passive (leak) conductance, a more metabolically economical approach would be to express voltage-activated K^+^ conductances that would make membrane more leaky only when it is needed [15, 18]. The downside is that the K^+^ conductances activate with an intrinsic delay but that plausibly can be managed by increasing the channels’ voltage sensitivity; alternatively, membrane charging can be sped up by increasing the channels’ expression.

Our discovery of a novel role for non-inactivating K^+^ conductances can be applicable to all above-described systems. However, the bandwidth-narrowing effect of the repolarizing K^+^ current is strictly parametric, apparently depending on the superposition of the depolarizing and repolarizing currents with the optimal delay. Because the benefits of bandwidth expansion can be realized without additional costs, simply by expressing a suitable K^+^ conductance, the phenomenon might be widespread. Also, it is possible that K^+^ conductances can modulate voltage responses to light even in the ciliary photoreceptors of vertebrates where phototransduction terminates in a transient closing of the cGMP-gated sodium channels, hyperpolarizing the cell [46], although that might require fine-tuning of K^+^ channel deactivation instead of activation kinetics.
